# Effects of Activated Cold Regenerant on Pavement Properties of Emulsified Asphalt Cold Recycled Mixture

**DOI:** 10.3390/ma18153529

**Published:** 2025-07-28

**Authors:** Fuda Chen, Jiangmiao Yu, Yuan Zhang, Zengyao Lin, Anxiong Liu

**Affiliations:** 1 School of Civil Engineering and Transportation, South China University of Technology, Guangzhou 510641, China; ctchenfd@163.com (F.C.); yujm@scut.edu.cn (J.Y.); 2 Central Fortune Creation Technology Group Co., Ltd., Foshan 528313, China; 19574488298@163.com; 3 Institute of Highway Engineering, RWTH Aachen University, 52074 Aachen, North Rhine-Westphalia, Germany; z.lin@isac.rwth-aachen.de

**Keywords:** cold recycled asphalt mixtures, emulsified asphalt, activated cold regenerant, pavement performance, cracking and fatigue resistance

## Abstract

Limited recovery of the viscoelastic properties of aged asphalt on RAP surfaces at ambient temperature reduces interface fusion and bonding with new emulsified asphalt, degrading pavement performance and limiting large-scale promotion and high-value applications of the emulsified asphalt cold recycled mixture (EACRM). Therefore, a cold regenerant was independently prepared to rapidly penetrate, soften, and activate aged asphalt at ambient temperature in this paper, and its effects on the volumetric composition, mechanical strength, and pavement performance of EACRM were systematically investigated. The results showed that as the cold regenerant content increased, the air voids, indirect tensile strength (ITS), and high-temperature deformation resistance of EACRM decreased, while the dry–wet ITS ratio, cracking resistance, and fatigue resistance increased. Considering the comprehensive pavement performance requirements of cold recycled pavements, the optimal content of the activated cold regenerant for EACRM was determined to be approximately 0.6%.

## 1. Introduction

Emulsified asphalt cold recycling is a sustainable pavement technology that utilizes emulsified asphalt, reclaimed asphalt pavement (RAP), water, and fillers as raw materials. Through ambient-temperature mixing, paving, and compaction, it enables the construction of recycled pavement structures. This technology is widely used for base course or lower surface layer construction, offering low energy consumption, reduced costs, and high resource utilization (up to 100%) [1,2]. Furthermore, it effectively addresses environmental issues from asphalt waste accumulation and reduces reliance on natural aggregates, offering significant economic and environmental benefits [3,4,5].

However, emulsified asphalt cold recycled mixture (EACRM) generally exhibits inferior performance compared to hot-mixed asphalt mixture in terms of mechanical strength, moisture resistance, and long-term durability, which limits its application in high-grade pavement or higher structural layers [1,6,7]. The fundamental challenge lies in the absence of external heating during cold recycling, which restricts the viscoelastic recovery of aged asphalt in RAP and weakens its bonding capacity with the newly added emulsified asphalt [8,9,10]. Previous studies show that aged asphalt’s high carbonyl and sulfoxide content, along with its physical hardening, reduce its wettability, diffusion, and fusion with new emulsified asphalt, which creates weak interfacial zones at the microscale, leading to premature cracking, water damage, and fatigue failure [11,12,13]. Although the problem can be partially solved by adding cement or optimizing gradation, it is difficult to effectively activate the bonding potential of aged asphalt and achieve deep integration with new asphalt [14,15].

To enhance the pavement performance and broaden the engineering applicability of EACRM, extensive research and practical applications have been conducted: (1) Over 80% of EACRM’s mechanical strength comes from emulsified asphalt. Using higher-viscosity emulsified asphalt can improve the fatigue resistance, crack resistance, and deformation resistance of EACRM [7,16]. (2) Adding fibers to EACRM creates an emulsified asphalt– fiber network that limits micro-crack growth, enhancing mechanical strength and crack resistance [17,18,19,20]. (3) Adding appropriate amounts of cementitious materials like fly ash, silica fume, or volcanic ash can increase the stiffness modulus, early strength, and water damage resistance of EACRM but may raise concerns about excessive heavy metal content [21,22,23,24]. (4) Adding early strength agents, wetting agents, or water reducers can promote rapid demulsification of emulsified asphalt and improve its coating uniformity on RAP. However, dosages should be controlled to avoid excessive demulsification, agglomeration, or uneven mixing, which could reduce pavement performance [25,26,27,28].

Although the mentioned methods have contributed to improving the pavement performance of EACRM, they provide limited enhancement to the physical and chemical properties of aged asphalt. Therefore, some studies have attempted to restore the activity of aged asphalt and enhance the interfacial fusion with new emulsified asphalt by adding waste vegetable oil, EVOTHERM-DAT, mineral oil, hot-mixed regenerants, or epoxy resin-based emulsions [29,30,31,32,33,34,35,36,37,38]. Relevant research has demonstrated that these materials can effectively improve the cracking resistance, moisture stability, and fatigue performance of cold recycled mixtures to varying extents, notably enhancing the flexibility of aged asphalt and interfacial bonding strength. However, these materials are mostly used in research and are difficult to apply in actual engineering due to the following reasons: (1) The main components of numerous related regenerators are petroleum distillation products, which may pose potential risks due to their excessive toxicity, strong corrosivity, or high flammability [39]. (2) Using waste vegetable oil as an activator requires pre-mixing with RAP and fails to be widely adopted due to issues such as space requirements, complex procedures, and difficulty in controlling the optimal pre-moistening time [34,37]. (3) Most hot-mixed rejuvenators have high viscosity, making uniform and rapid coating with RAP at ambient temperature difficult and causing significant fluctuations in material performance. In addition, it takes a long time to soften and activate the aged asphalt, affecting the long-term performance of cold recycled pavement [40,41,42].

Consequently, it is essential to develop a new cold regenerant with rapid penetration, ambient-temperature activation efficacy, and environmental friendliness to solve the technical challenge of activating aged asphalt in cold recycling, which potentially enables a transition from “physical coating” to “adsorptive integration” at the interface between new and aged asphalt and releases the performance potential of EACRM. The research is significant for expanding cold recycling applications, enhancing low-carbon pavement technology reserves, and promoting an environmentally friendly society.

## 2. Materials and Mixture Design

### 2.1. Raw Materials

#### 2.1.1. RAP

The RAP was sourced from the reclaimed SBS modified asphalt mixture collected from the upper surface layer of a municipal road in Foshan, Guangdong province. The material was finely crushed and sieved into three particle size ranges: 0–8 mm, 8–13 mm, and 13–20 mm. Before specimen preparation, the RAP was dried in an oven at 60 °C for 24 h to eliminate moisture, thereby negating the need for further moisture content testing. The technical properties of the RAP are summarized in Table 1.

#### 2.1.2. Modified Emulsified Asphalt

The modified emulsified asphalt was provided by Central Fortune Creation Technology Group Co., Ltd., Foshan, China. The emulsion contains 54–56% base asphalt (Pen 60–70), 35–37% water, 5~6% Styrene-butadiene-styrene block copolymer, 2.0–2.5% cationic surfactant, 0.4–0.5% polyvinyl acetate stabilizer, and 0.2–0.3% aluminum silicate by weight. Its technical properties are shown in Table 2.

#### 2.1.3. Aggregates and Mineral Fillers

The new aggregates consist primarily of 0–3 mm diabase manufactured sand from Furong Quarry in Heyuan, Guangdong. The filler is limestone mineral powder produced by Quanfa Stone Factory in Foshan, Guangdong. The cement is P.O 42.5 Portland cement produced by Yingde Conch Cement Co., Ltd. The technical properties of the new aggregates and powder are shown in Table 3 and Table 4.

#### 2.1.4. Activated Cold Regenerant

The composition of the activated cold regenerant developed by the research group includes the following:

(1) 40–60% bio-based solvents (primarily plant-derived terpenes and/or fatty acid esters) for aged asphalt dissolution and penetration, which are naturally derived from plants and possess non-toxic and biodegradable properties.
(2) 25–40% short-chain alcohol solvents to regulate volatility and dispersibility.
(3) 10–20% lipophilic nonionic surfactants to promote stable oil–water interfaces by reducing interfacial tension.
(4) 10–15% deionized water to control viscosity and homogeneity.

It features low viscosity and water-like fluidity, enabling rapid penetration into aged asphalt at ambient temperature, thereby promoting its softening, activation, and interfacial fusion with newly added emulsified asphalt. Compared to traditional rejuvenators, the activated cold regenerant has low toxicity, high biodegradability, and no environmental pollution or negative effects on human health. The technical properties of the activated cold regenerant are summarized in Table 5.

**Figure 1:**
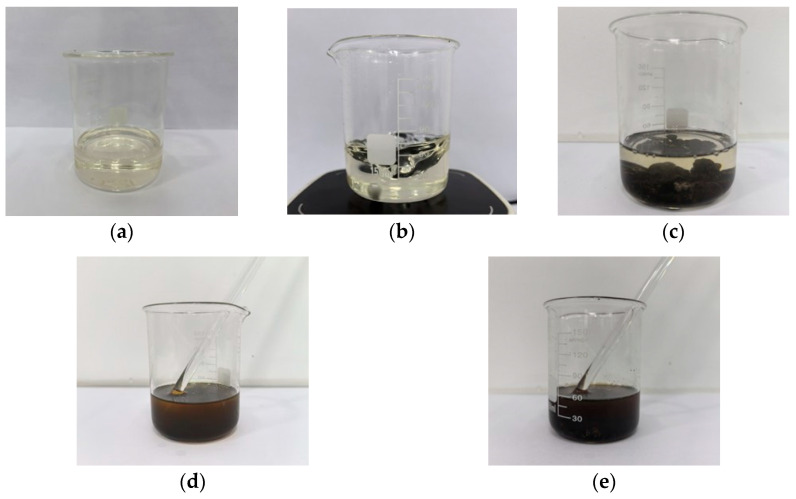
Physical properties and activation effect of the activated cold regenerant. (**a**) Appearance; (**b**) Fluidity under magnetic stirring; (**c**) RAP immersed in the regenerant; (**d**) After 1 min of stirring; (**e**) After 30 min of standing.

As illustrated in Figure 1, the regenerant appears as a clear, free-flowing liquid. Upon immersing RAP into the regenerant, aged asphalt begins to dissolve from the aggregate surface. After 1 min of stirring, the regenerant becomes visibly turbid and tan-colored, indicating the initial activation and dispersion of aged asphalt. After 30 min of standing, the solution turns darker and opaque, confirming the continued dissolution and interaction process.

### 2.2. Design of EACRM

#### 2.2.1. Gradation Design

Based on the particle size composition of raw materials and gradation design criteria, the proportions of RAP, aggregates, and fillers, as well as the synthetic gradation, were determined as shown in Table 6. Additionally, 5000 g of RAP was prepared according to the synthetic gradation, and the asphalt content was measured to be 4.05% by extraction.

#### 2.2.2. Optimal Moisture Content and Modified Emulsified Asphalt Content Determination

The EACRM mixing procedure consists of four sequential steps. First, RAP, manufactured sand, and water were mixed for 150 s. Next, modified emulsified asphalt was added and mixed for an additional 90 s. Subsequently, mineral filler and cement were introduced and blended for another 90 s. When the activated cold regenerant was used, it was evenly poured onto the RAP and pre-stirred for 60 s to achieve surface coating of all RAP particles, after which the remaining materials were added in the same sequence. The mixing temperature was controlled at 25 ± 2 °C.

1. Determination of optimal moisture content

**Figure 2:**
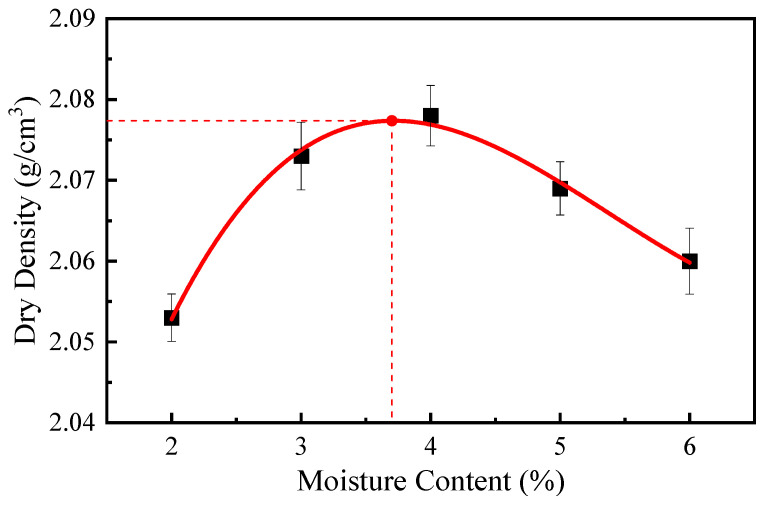
Test results of heavy compaction tests.

The dosage of modified emulsified asphalt was fixed at 4.0%, the cement content at 1.0%, and the moisture content varied from 2.0% to 6.0%. The EACRM was compacted in three layers using the heavy compaction method, with each layer compacted 98 times. After compaction, the dry density of each group was calculated as shown in Figure 2. Based on these results, the optimal moisture content for the EACRM was determined to be 3.7%.

2. Determination of optimal dosage of modified emulsified asphalt

**Figure 3:**
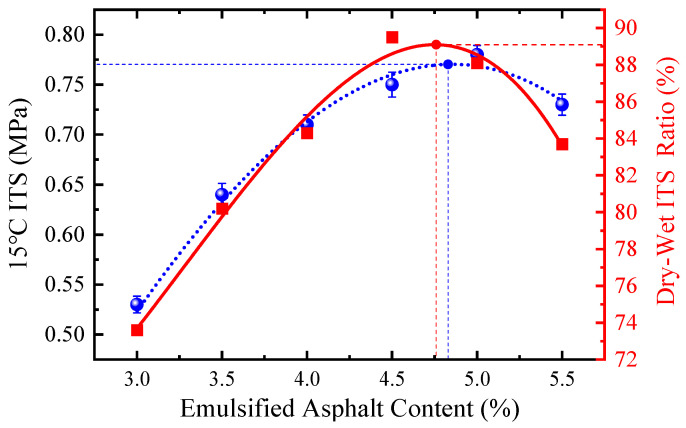
Test results of 15 °C ITS and dry–wet ITS ratio.

The optimal moisture content and cement content were fixed at 3.7% and 1.0%, respectively, while the dosage of modified emulsified asphalt was varied from 3.0% to 5.5%. The mixed EACRM was placed into Marshall molds and compacted 50 times on each side in accordance with the Marshall test method. The specimens were then cured in an oven at 60 °C for 48 h without demolding, followed by an additional compaction of 50 times on each side. Finally, the specimens were cooled at room temperature for 12 h before demolding. The results of 15 °C indirect tensile strength (ITS) and dry–wet ITS ratio of different groups are shown in Figure 3. Based on these results, the optimal dosage of modified emulsified asphalt was determined to be 4.8%.

After determining the optimal moisture content and the optimal dosage of modified emulsified asphalt, the activated cold regenerant was added into the EACRM at dosages of 0.3%, 0.6%, and 0.9% by weight. It was discovered that the activated cold regenerant exhibited water-like workability during mixing. Therefore, when adding the activated cold regenerant, seven experimental groups with maintained moisture content or reduced moisture content (reducing the same volume of water) were established as shown in Table 7.

## 3. Test Methods and Evaluation Indicators of EACRM

### 3.1. Volume Index Measurement

Since the water absorption of EACRM specimens generally exceeds 2%, the bulk specific gravity was determined using the wax-sealing method. The theoretical maximum density was measured via the vacuum method to simulate ideal compaction conditions, thereby minimizing the influence of material heterogeneity and internal micro-voids on the density results. Subsequently, the air void content was calculated based on the ratio of bulk specific gravity to theoretical maximum density.

### 3.2. Indirect Tensile Strength Test (ITS)

The ITS test was adopted to analyze the strength variation in different groups of EACRM. Specimens from different groups were randomly divided into two subgroups, with each subgroup containing at least four specimens. One subgroup of specimens was fully immersed in a 15 °C water bath for 2 h, then tested to obtain 15 °C ITS. Another subgroup of specimens was first immersed in a 25 °C water bath for 22 h, then in a 15 °C water bath for 2 h, and tested to obtain ITS after 24 h of immersion. Finally, the dry–wet ITS ratio was calculated as the ratio of the ITS after 24 h of immersion to 15 °C ITS.

### 3.3. Indirect Tensile Asphalt Cracking Test (IDEAL-CT Test)

**Figure 4:**
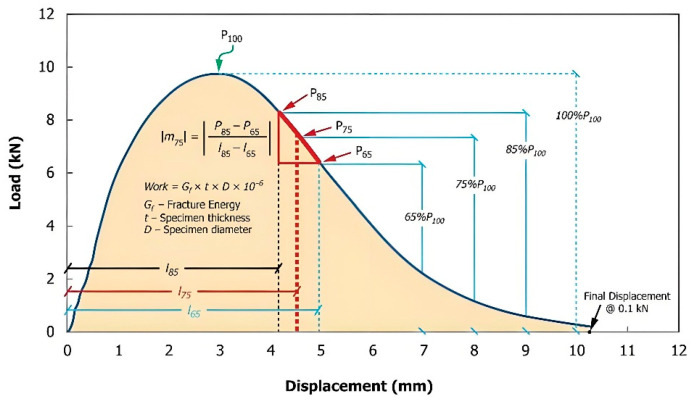
Typical load–displacement curve of IDEAL-CT test [44].

The IDEAL-CT test was conducted to assess the cracking resistance of EACRM. According to ASTM D8225-2019 [44], the test fixture was chosen with 2 cm clamping strips. The load was applied along the specimen diameter, and the test temperature was set at 15 °C and 25 °C. Before testing, the specimens were conditioned in the environmental chamber at the respective test temperatures for over 4 h. The loading rate was controlled at 50 mm/min, and the test was terminated when the applied force dropped below 0.1 kN. A representative load–displacement curve obtained from the IDEAL-CT test is shown in Figure 4.

The CT_index_ is the key evaluation index in the IDEAL-CT test, calculated as shown in Equation (1). A higher CT_index_ indicates better crack resistance. In Equation (1), G_f_ refers to the fracture energy per unit cracking area, representing the resistance capacity of the EACRM during loading, and |m_75_| refers to the absolute slope value at the point where the peak force decreases to 75%, representing the crack propagation rate of the EACRM. The calculation of Gf and |m_75_| is shown in Equations (2) and (3), respectively.

(1) $${\mathrm{CT}}_{\mathrm{index}}=\frac{t}{62}\frac{l_{75}}{D}\frac{G_{f}}{\left|m_{75}\right|}\times 10^{6}$$

(2) $$G_{f}=\frac{W}{\mathrm{Dt}}\times 10^{6}=\frac{\int \mathrm{Pdl}}{\mathrm{Dt}}{\times 10}^{6}$$

(3) $$\left|m_{75}\right|=\left|\frac{P_{85}-P_{65}}{I_{85}-I_{65}}\right|$$

where t is the thickness of the specimen; D is the diameter of the specimen; P_85_ and P_65,_, respectively, refer to the 85% peak force and 65% peak force; and I_85_ and I_65_, respectively, refer to the displacements when the peak force decreases to 85% and 65%.

### 3.4. Indirect Tensile Fatigue Test

The stress-controlled indirect tensile fatigue test was conducted to evaluate the fatigue resistance of EACRM. The test applied a periodic vertical load to the Marshall specimens, generating horizontal tensile stress and causing fatigue damage in EACRM. The test temperature was set at 15 °C with the test frequency of 2 Hz. The test load was set by various stress ratios (0.2, 0.3, 0.4, and 0.5.) which is defined as the loading stress divided by the indirect tensile strength at 15 °C. Each loading cycle included 100 ms of loading and 400 ms of unloading. The fatigue failure was defined as the formation of a through-crack in the specimen, and the corresponding number of cycles at failure was recorded as the fatigue life.

### 3.5. Hamburg Wheel Tracking Test

The Hamburg wheel tracking test was adopted to evaluate the high-temperature stability of EACRM. According to AASHTO T324 [45], the water bath temperature was set at 50 °C. During the test, a steel wheel was used to apply reciprocating loading to the EACRM of different groups. The test stopped when the loading frequency reached 20,000 cycles or the rutting depth reached 20 mm. The high-temperature stability of the mixture is characterized by the creep resistance rate (Cr) and the total deformation rate (Dr). Cr indicates the number of rolling passes required for 1 mm of rutting in the mixture, and Dr is the ratio of post-test rutting depth to total test duration. A mixture with higher Cr and lower Dr shows better high-temperature deformation resistance and less rutting under hydrothermal effect.

## 4. Results and Discussion

### 4.1. Volume Indicators

**Figure 5:**
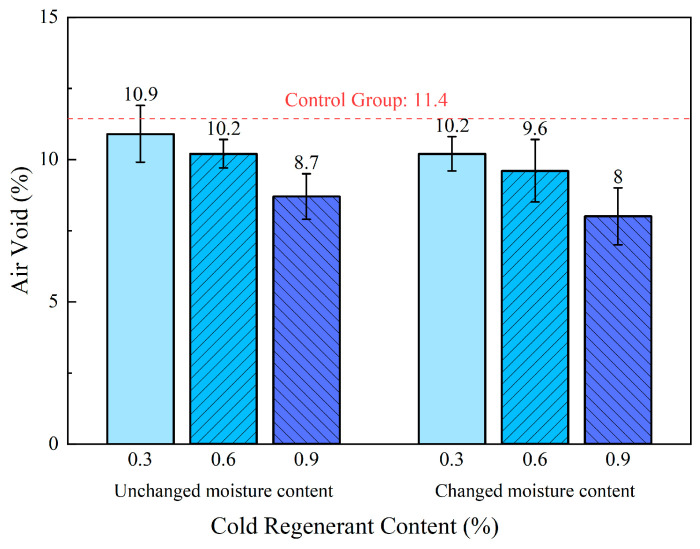
Effect of activated cold regenerant content on air void.

As shown in Figure 5, the air void contents of EACRM mixtures were measured and calculated for different groups. The incorporation of activated cold regenerant led to a decrease in air void content compared to the control group, with further reductions observed as the regenerant dosage increased. This trend aligns with improved wetting and potential softening of aged asphalt, promoting the particle rearrangement, and resulting in a denser internal structure.

Furthermore, the groups with reduced optimum moisture content had lower air voids than those with unchanged moisture content when the same cold regenerant content was added. This indicates that the cold regenerant effectively replaces water to provide lubrication. If the moisture content remains unchanged after adding the cold regenerant, excess water will hinder aggregate interlocking and weaken compaction. Additionally, the evaporation of surplus moisture can generate micro-voids, further contributing to an increase in the total air void content of the EACRM.

### 4.2. 15 °C ITS and Dry-Wet ITS Ratio

**Figure 6:**
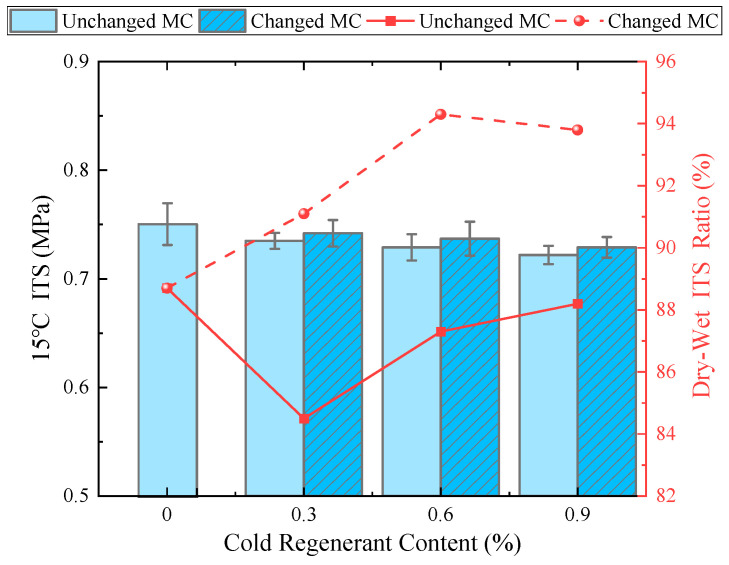
Effect of cold regenerant content on 15 °C ITS and dry–wet ITS ratio.

As shown in Figure 6, the 15 °C ITS and dry–wet ITS ratio for different EACRM specimens were measured. The column chart illustrates that the addition of cold regenerant led to a decrease in 15 °C ITS. As the cold regenerant content increased, the 15 °C ITS decreased correspondingly. However, when the content was limited to 0.9%, the impact on the 15 °C ITS was minimal, with a reduction of less than 4%. In addition, the groups with reduced optimum moisture content had higher 15 °C ITS than those with unchanged moisture content when the same cold regenerant was added.

Moreover, the point-line chart shows that adding the cold regenerant and varying the optimal moisture content significantly affected the dry–wet ITS ratio of the EACRM. When the optimal moisture content remained unchanged, the dry–wet ITS ratio of the EACRM with cold regenerant is lower than that of the control group. This reduction may be attributed to the excessive moisture impeding the fusion and bonding between the activated aged asphalt and the new emulsified asphalt during curing. In contrast, when an equivalent volume of water was reduced upon adding the regenerant, the dry–wet ITS ratio gradually increased with higher regenerant contents. When the content reaches 0.6%, the dry–wet ITS ratio peaks at 94.3% and then slightly declines. This shows that with an appropriate moisture content, adding the cold regenerant can effectively activate aged asphalt, improve fusion and adhesion with new emulsified asphalt, and enhance the water stability of the EACRM.

Based on Section 4.1 and Section 4.2, reducing the optimum moisture content after adding the activated cold regenerant is beneficial for decreasing the air void and enhancing the 15 °C ITS and dry–wet ITS ratio of the EACRM. Consequently, all the test groups in subsequent experiments reduced the same volume of water after adding the cold regenerant.

### 4.3. CT Index

**Figure 7:**
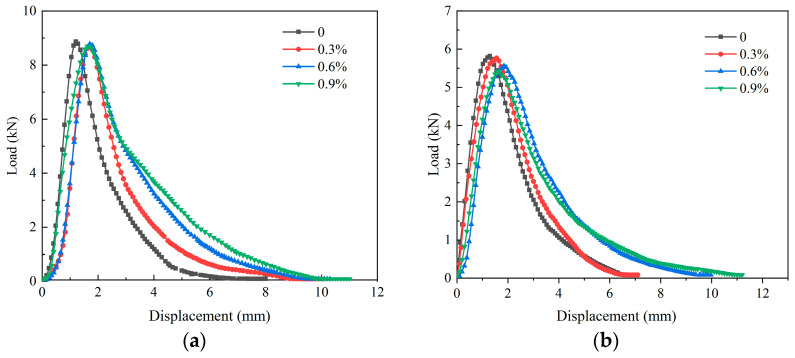
IDEAL-CT typical displacement–load plots of the mixture at different activated cold regenerant dosages. (**a**) 15 °C; (**b**) 25 °C.

The IDEAL-CT load–displace curves of EACRM with different cold regenerant content (0%, 0.3%, 0.6%, 0.9%) at 15 °C and 25 °C are shown in Figure 7. The peak load gradually decreased with the increase in cold regenerant content at either 15 °C or 25 °C, but the overall decline remained within 10%. The displacement at peak load increased with higher regenerant contents and reached a maximum at 0.6%, indicating that mixtures with 0.6% regenerant exhibited superior initial cracking resistance compared to the other groups.

**Figure 8:**
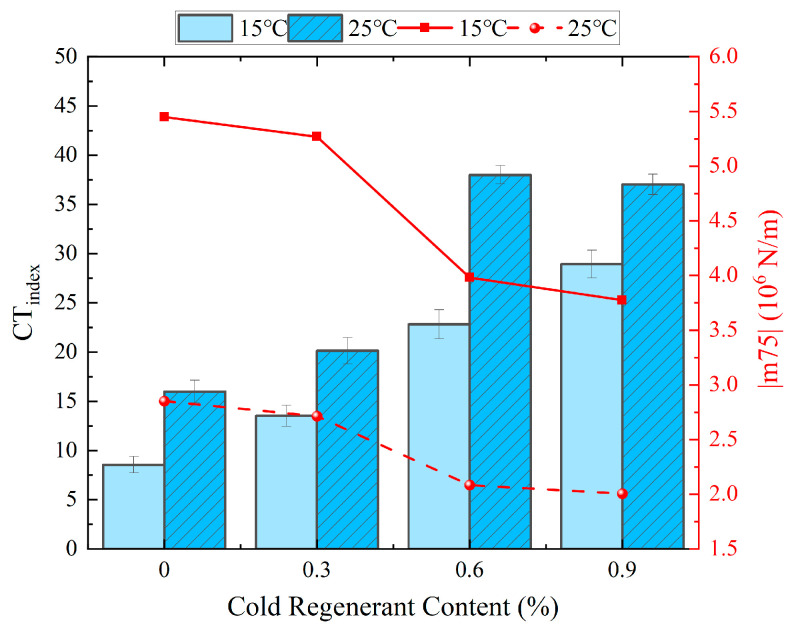
Effect of cold regenerant content on |m_75_| and CT_index_.

Figure 8 illustrates the CT_index_ and |m_75_| value calculated by Equations (1)–(3), quantitatively demonstrating the influence of cold regenerant content and temperature on the cracking resistance of EACRM: The CT_index_ gradually increased and |m_75_| continuously decreased with the increase in cold regenerant and temperature. At both 15°C and 25 °C, as the cold regenerant content increased from 0.3% to 0.6%, the CT_index_ sharply increased while |m_75_| sharply decreased. When the content further increased to 0.9%, the changes in CT_index_ and |m_75_| were insignificant or slightly reduced.

In the combined results from Figure 7 and Figure 8, it can be found that the cold regenerant effectively enhanced the plastic deformation capacity of EACRM and slowed crack expansion but required optimal content control. An insufficient amount of cold regenerant failed to activate aged asphalt or enhance interface fusion between new and aged asphalt, preventing improvement in cracking resistance of EACRM. Adding excessive cold regenerant delayed cracking development but reduced mechanical strength excessively, leading to worse cracking resistance.

### 4.4. Indirect Tensile Fatigue Life

Table 8 presents the indirect tensile fatigue life of EACRM mixtures with different cold regenerant content (0%, 0.3%, 0.6%, 0.9%) under different stress ratios. The cold regenerant rose to 0.6% and 0.9%, with a more significant increase at lower stress ratios (0.2 and 0.3), where improvements ranged from 6.6% to 31.7%. In combination with the air voids and dry–wet ITS ratio results in Section 4.1 and Section 4.2, it can be inferred that the addition of the cold regenerant enhanced interfacial bonding between aged asphalt and new emulsified asphalt, reduced air voids in EACRM, and inhibited fatigue damage caused by crack expansion along weak interfaces or internal defects (e.g., minute interconnected voids), thereby enhancing the fatigue performance.

However, at a regenerant content of 0.3%, the indirect tensile fatigue life of the EACRM was lower than that of the control group. A plausible explanation is that insufficient cold regenerant only partially softened the aged asphalt surface layer, leading to discontinuous interface fusion between the incompletely activated aged asphalt and the emulsified asphalt. The reduced interfacial bonding strength made the interface a preferred path for fatigue failure, ultimately decreasing the fatigue performance of the mixture.

### 4.5. Cr and Dr

**Figure 9:**
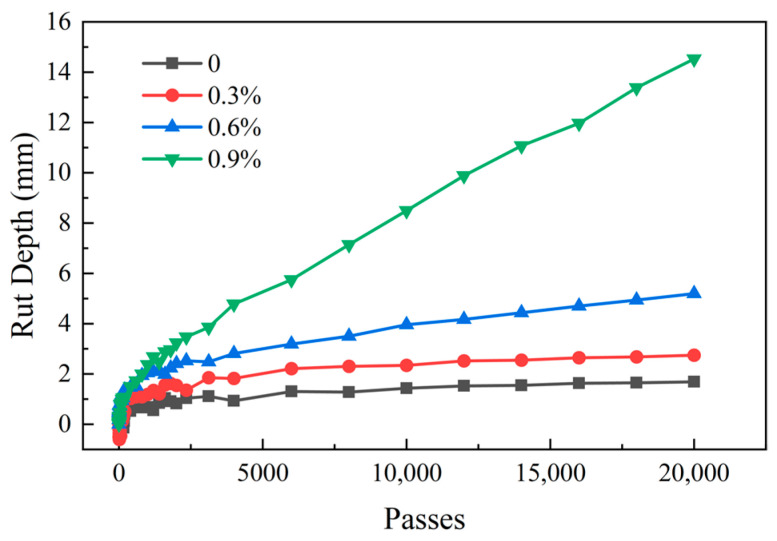
Hamburg rutting curves of EACRM with different cold regenerant content.

As shown in Figure 9, the high-temperature performance of EACRM with different cold regenerant content in the Hamburg wheel tracking test is inferior to that of the control group, and their high-temperature deformation resistance decreased as the cold regenerant content increased.

**Figure 10:**
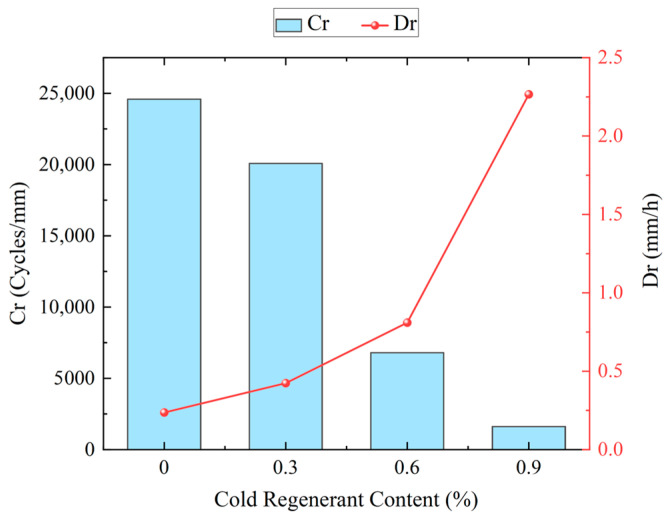
Variation in high-temperature performance of EACRM with different cold regenerant content.

Figure 10 shows that when the cold regenerant content reached 0.3%, the high-temperature deformation resistance of the EACRM was similar to that of the control group; when the cold regenerant content reached 0.6%, the Cr dropped significantly to about 6800 times/mm but the Dr remained low, indicating reduced creep resistance but satisfactory deformation resistance; when the cold regenerant content reached 0.9%, the Dr increased significantly and the Cr was lowest. These phenomena suggested that a high dosage of cold regenerant severely softens aged asphalt, significantly affecting the deformation resistance of EACRM under hydrothermal conditions. Additionally, it can be found that none of the Hamburg rutting curves showed significant slope changes after 20,000 times of wheel rolling in 50 °C water bath, indicating that all four EACRMs had excellent water damage resistance, with stripping points exceeding 20,000 cycles.

## 5. Conclusions

In this research, an activated cold regenerant was independently developed to rapidly penetrate, soften, and activate aged asphalt at ambient temperature. Its effects on the volumetric composition, mechanical strength, and pavement performance of EACRM were systematically investigated. The main conclusions are as follows.

(1) As the cold regenerant content increases, the air voids, 15 °C ITS, and high-temperature deformation resistance decrease, while the dry–wet ITS ratio, cracking resistance, and fatigue performance improve. This indicated that the cold regenerant potentially softens aged asphalt and enhances its wettability, and improves the coating and interfacial adhesion between new and aged asphalt, which is conducive to densifying the internal structure under compaction and enhancing the crack resistance, fatigue resistance, and water damage resistance of EACRM, but reduces its stiffness and high-temperature performance partially.
(2) At a dosage of 0.3%, the EACRM showed minor differences in air voids, 15 °C ITS, dry–wet ITS ratio, cracking resistance, and high-temperature performance compared to the control group. Its fatigue performance was inferior, indicating insufficient activation of aged asphalt by the cold regenerant. At a dosage of 0.6%, the air voids decreased significantly; the dry–wet ITS ratio, cracking resistance, and fatigue performance were superior, with minor differences compared to the 0.9% dosage group. Although the aged asphalt was softened and caused a notable decline in creep deformation resistance compared to the control group, its Cr reached 6800 times/mm and its Dr remained low, indicating satisfactory rutting deformation resistance. At a dosage of 0.9%, the EACRM has minimum air voids and optimal water damage, cracking, and fatigue resistance. However, excessive cold regenerant severely softened aged asphalt, significantly deteriorating high-temperature performance and presenting a risk of insufficient deformation resistance.
(3) In summary, 0.6% is recommended as the optimal dosage of the activated cold regenerant in this study, striking a balance between enhanced cracking/fatigue resistance and acceptable high-temperature performance. It requires calibration according to RAP characteristics, grading design, and moisture content in actual applications to meet the requirements for anti-rutting performance, cracking resistance, and water stability, while considering its cost-effectiveness.
(4) This study primarily focused on the macroscopic performance of EACRM rejuvenated by the activated cold regenerant, including volumetric composition, mechanical strength, and pavement properties. Future works will investigate the rheological behavior of aged asphalt rejuvenated by the activated cold regenerant (e.g., complex modulus, phase angle) and its interfacial microstructure evolution (e.g., via SEM, AFM, or molecular dynamics simulation), ultimately establishing a multi-scale mechanism linking physicochemical changes to engineering performance.

## Figures and Tables

**Table 1:** Technical properties of RAP.

Test Items	Requirements	Results	Test Method
Maximum particle size of RAP (mm)	≤19.0	≤19.0	JTG/T 5221 Appendix B [43]
Sand equivalent of 0–8 mm RAP (%)	≥50	78.3	JTG/T 5221 Appendix B [43]
25 °C Penetration of aged asphalt (0.1 mm)	≥10	16.5	JTG E20 T 0604

**Table 2:** Technical properties of modified emulsified asphalt.

Test Items		Requirements	Results	Test Method
Setting rate		Slow-setting	Slow-setting	JTG E20 T 0658
Residue on 1.18 mm sieve (%)		≤0.1	0.1	JTG E20 T 0652
Particle charge		Cation (+)	Cation (+)	JTG E20 T 0653
Viscosity, Saybolt Furol at 25 °C (s)		7~100	65	JTG E20 T 0623
Evaporation Residue	Residue content (%)	≥60	63.2	JTG E20 T 0651
	25 °C Penetration (0.1 mm)	50~130	53.4	JTG E20 T 0604
	Softening point (°C)	/	82.5	JTG E20 T 0606
	5 °C Ductility (cm)	≥20	42	JTG E20 T 0605
	Solubility in trichloroethylene (%)	≥97.5	99.0	JTG E20 T 0607
	25 °C Elastic recovery (%)	/	93.4	JTG E20 T 0662
Adhesion to coarse aggregate (Coating area)		≥2/3	≥2/3	JTG E20 T 0654
Mix test with aggregate		Uniform	Uniform	JTG E20 T 0659

**Table 3:** Technical properties of manufactured sand.

Test Items	Requirements	Results	Test Method
Apparent relative density	≥2.50	2.778	JTG 3432 T 0328
Soundness (>0.3 mm) (%)	≤12	8.0	JTG 3432 T 0340
Sand equivalent (%)	≥65	72.0	JTG 3432 T 0334

**Table 4:** Technical properties of powder.

Test Items	Requirements	Results	Test Method
Apparent density	≥2.50	2.794	JTG 3432 T 0352
Moisture content (%)	≤1	0.2	JTG 3432 T 0359
Hydrophilic coefficient	<1	0.7	JTG 3432 T 0353
Plasticity index (%)	<4	2.8	JTG 3432 T 0354

**Table 5:** Physical properties of the activated cold regenerant in this study.

Test Items	Results	Test Method
Appearance	Transparent and slightly yellow liquid	Visualization
Density (g/cm^3^)	0.84	Bottle method
Rotational viscosity at 25 °C (mPa·s)	92 (at 50 s^−1^)	JTG E20 T 0625
Flash point (°C)	82	ASTM D93

**Table 6:** Synthetic gradation of EACRM.

Sieve Size (mm)	The Mass Percentage Passing Through the Specific Sieve (%)							
	RAP			Sand	Powder	Cement	Gradation	Design Range
	13~20 mm	8~13 mm	0~8 mm					
26.5	100.0	100.0	100.0	100.0	100.0	100.0	100.0	100.0
19	87.3	100.0	100.0	100.0	100.0	100.0	95.9	90~100
9.5	4.5	43.4	95.9	100.0	100.0	100.0	58.8	50~75
4.75	0.3	19.0	70.4	100.0	100.0	100.0	43.3	26~60
2.36	0.3	8.3	38.4	91.3	100.0	100.0	28.1	20~45
0.3	0.3	0.8	5.3	32.1	100.0	100.0	8.9	5~18
0.075	0.3	0.4	2.3	12.4	89.2	98.4	5.7	4~10
Proportion (%)	32.0	16.0	40.0	8.0	3.0	1.0	—	—

**Table 7:** Summary of test groups.

Test Groups		Cold Regenerant Content (%)	Emulsified Asphalt Content (%)	Moisture Content (%)
Control group	1	0	4.8	3.70
Maintained optimal moisture content	2	0.3	4.8	3.70
	3	0.6	4.8	3.70
	4	0.9	4.8	3.70
Reduced optimal moisture content	5	0.3	4.8	3.33
	6	0.6	4.8	3.05
	7	0.9	4.8	2.58

Remarks: The dosage of modified emulsified asphalt, water, and activated cold regenerant denotes the weight ratio relative to RAP and aggregates.

**Table 8:** Indirect tensile fatigue life of cold recycled mixtures with different cold regenerant content.

Cold Regenerant Content (%)	Stress Ratio			
	0.2	0.3	0.4	0.5
0	65,000	12,000	2800	760
0.3	58,000	10,000	2900	680
0.6	69,000	14,000	3400	740
0.9	85,000	15,000	3300	800

## Data Availability

The original contributions presented in this study are included in the article. Further inquiries can be directed to the corresponding author(s).
